# Reliability and Validity of the Osteoarthritis Research Society International Minimal Core Set of Recommended Performance-Based Tests of Physical Function in Knee Osteoarthritis in Community-Dwelling Adults

**DOI:** 10.21315/mjms2020.27.2.9

**Published:** 2020-04-30

**Authors:** Ariyachaikul Suwit, Kanthain Rungtiwa, Thonglorm Nipaporn

**Affiliations:** Department of Physical Therapy, Faculty of Associated Medical Sciences, Chiang Mai University, Thailand

**Keywords:** osteoarthritis, knee, performance-based test, physical function, outcomes, reliability, validity

## Abstract

**Background:**

The proper reliability analysis for specific type of data and limit study of various types of construct validity are crucial for performance-based tests for the knee osteoarthritis (OA) population. The purpose of this study was to evaluate relative and absolute reliability and construct validity of the Osteoarthritis Research Society International (OARSI) recommended minimal core set of performance-based tests in knee OA in community-dwelling adults.

**Methods:**

Fifty-five primary knee OA (median age 69.0, interquartile range [IQR] 11.0) participated in the cross-sectional study. Three performance-based tests were performed in two sessions with a 1-week interval; 30-s chair stand test, 40-m fast-paced walk test and 9-step stair climb test. Relative reliability included intra-class correlation and Spearman’s correlation coefficient (SPC). Absolute reliability included standard error of measurement, minimum detectable change, coefficient of variance, limit of agreement (LOA) and ratio LOA. Knee Injury and Osteoarthritis Outcome Score-Physical Function Short Form (KOOS-PS), knee extensor strength and pain scale were analysed for convergent validity using Pearson’s correlation coefficient and SPC. Analysis of Covariance was utilised for known-groups validity.

**Results:**

Relative and absolute reliability were all acceptable. LOA showed small systematic bias. Acceptable construct validity was only found with knee extensor strength. All tests demonstrated known-groups validity with medium to large effect size.

**Conclusion:**

The OARSI minimum core set of performance-based tests demonstrated acceptable relative and absolute reliability and good known-groups validity but poor convergent validity.

## Introduction

Knee osteoarthritis (OA) is the deteriorative risk to locomotive function and consequential frailty (1–2). Feasible and evidence-based measurement tools were needed for early detection and follow up of the disease state (3). Assessment of physical function in knee OA is classified into self-reported and performance-based tests that conceptualised on the International Classification of Functioning, Disability and Health (ICF) (3–4). The minimal core set of three performance-based tests were recommended by the Osteoarthritis Research Society International (OARSI), which includes 30-s chair stand test (30sCST), 40-m fast-paced walk test (40mFPWT) and stair climb test (SCT) (3, 5). The 30sCST was preferred because of no floor effect in which poor physical function could complete the test (6). Forty-metre fast-paced walk test, a time-based, short-distance, untimed-turn and maximum walk speed, is a good test to evaluate performance response with environmental demand and is appropriate for lower extremity OA (7–8). Stair negotiation is one of the most difficult tasks to overcome. It is associated with the limit of function from sarcopenia, somatosensory and visual impairment and other disorders such as OA (9).

The assessment of psychometric property of the test is specific to clinical conditions and should cover various reliability (absolute and relative reliability) and construct validity (e.g. convergent, divergent and known-group) (4, 10–11). Two measurement property studies that targeted the OARSI recommended minimal core set of three performance-based tests in knee OA and knee arthroplasty (12–13). One of those studies showed acceptable relative reliability of all performance-based tests except 11-step SCT in which the outlier was removed before analysis (12). Moreover, in non-normal data of 11-step SCT (outlier not removed), minimum detectable change (MDC) was not calculated (12). It was contended that human performance measurement, which uses ratio scale, tended to be heteroscedastic (i.e. departure from normality; error related with measurement value) (10, 14). In this heteroscedastic type of data, the standard error of measurement (SEM) is a less effective representation of measurement error than the coefficient of variance (CV) and ratio limit of agreement (ratio LOA) (10, 15). So, the proper analysis for a specific type of data is much more concerned with reliability study. Another psychometric property study of the OARSI recommended minimal core set showed that three performance-based tests had a moderate correlation with quadriceps strength and low correlation with a self-reported test (Knee Injury and Osteoarthritis Outcome Score-Physical Function Short Form [KOOS-PS]) and pain scale (13). Although this study provided a thorough convergent validity analysis, it did not investigate known-group validity. In a known-group study of 30sCST, the participants who ambulated with an assistive device were significantly different from the ones who did not (16). Therefore, the comparative study of the measurement property of comprehensive OARSI recommended minimal core set of performance-based tests were still needed (5). The purpose of this study was to evaluate the relative and absolute reliability and construct validity of the OARSI recommended minimal core set of performance-based tests in knee OA in community-dwelling adults.

## Methods

### Population and Sampling

This cross-sectional study of construct validity and reliability of three recommended performance tests for knee OA was performed in a sub-district community setting of Chiang Mai, a northern province of Thailand. The sample size for both construct validity and reliability was at least 50, as stated elsewhere (17). The study project was advertised via the community volunteer, the village head man and the Sub-district Health Promoting Hospital (SHPH) personnel. The volunteers were screened by physical therapists and were included if they (i) met the American College of Rheumatology (ACR) clinical diagnostic criteria (with classification tree) for knee OA (18–19); (ii) were able to follow the instructions and could perform sit-to-stand, walk and climb the stairs and (iii) could read and fill out the questionnaire by themselves or with the help from their relatives who will read for them.

Subjects were excluded if they had a history of (i) rheumatoid and gouty arthritis or secondary OA; (ii) pain on the lower back and lower extremities other than the knees; (iii) injury/fracture with/without surgery on the lower back and lower extremities; (iv) congenital or acquired anomalies in the spine and lower extremities; (v) neurological problems; (vi) heart disease or high blood pressure not controlled by medication; (vii) intra-articular injection within 3 weeks; (viii) receiving alcohol or medications within 24 h that affect sleep or (ix) hearing and visual loss.

## Procedures

Participants were appointed at the SHPH for the data collection on two sessions of performance-based testing, 1 week apart, to minimise recall effect and ensure real performance change (12). In session 1, participants performed three performance-based tests (30sCST, 40mFPWT and 9-step SCT) with three independent raters, i.e. each rater rated all participants by using only one test. All raters were physical therapists who had 7–12 years of clinical experience. The testing order of the tests for each participant was randomised to prevent a carry-over effect. A 5-min rest period between two consecutive measurements was allowed to ensure energy recruitment and fatigue prevention. Before testing, the physical therapist that was responsible for a specific test demonstrated the task, allowed the subjects to follow, gave feedback and stayed for safety prevention. In the first session, other than performance-based tests, the following data were collected: (i) baseline demographics; (ii) level of knee pain experienced over the past week assessed by an 11-point pain numerical rating scale (NRS) with 0 = no pain and 10 = worst pain (20); (iii) self-report difficulty to perform physical function assessed by the KOOS-PS and (iv) isometric knee extensor force measured with a hand-held dynamometer (HHD). In the second session, participants repeated three performance-based tests in the same order as they had done in the first session with the same raters. All raters were blind from the outcome of the first session. Half-day training for the test-specific therapists was taken before the data collection of the first week, including the following: questionnaire completion, NRS and KOOS-PS; set up, administration and recording of HHD and performance-based tests.

## Performance-Based Tests

Measurement of the OARSI recommended minimal core set of performance-based tests (30sCST, 40mFPWT and 9-step SCT) were strictly administered with the standard procedures provided in the OARSI website (https://www.oarsi.org/research/physical-performance-measures). The 30sCST was performed on a chair, 43 cm in height, with straight backrest and without armrest. The 40mFPWT was performed outdoors by walking straight at a distance of 10 m four times. Fast walking speed was calculated by excluding the turning time. The 9-step SCT was performed in the SHPH building on a 9-step (19 cm height/step) flight of stairs with handrail. The participants were allowed to use ambulation aids and/or handrail during the walking and stair-climbing tests.

## KOOS-PS

KOOS-PS is a 7-item self-report on each individual’s difficulty in performing daily functions. The 5-point Likert scale, which ranged from no difficulty to extreme difficulty, was rated. The scale was developed by using the Rasch analysis of multiple samples from many countries and extracted only seven most valid items (21). KOOS-PS showed good internal consistency (0.89) and good test–retest reliability (0.85–0.86) (22). Thai-version KOOS reported good internal consistency (0.9) and high test–retest reliability in ADL domains and moderate correlation to aggregated functional performance time (0.38 to 0.50) (23).

## Isometric Knee Extensor Torque

The maximum voluntary isometric contraction was tested with Baseline^®^ Hydraulic Hand Dynamometer (Fabrication Enterprise Inc., Elmsford, NY, USA). The dynamometer is liquid-hydraulic, 683 g in weight and is able to measure up to a maximum of 90 kg. An adaptor was fixed with the distal leg just above the lateral malleolus (24). The participant sat upright on the table with the arms crossed and the hip and knee flexed at 90° (0° as full knee extension) (24). Each participant performed two maximal contractions with a 5-min rest interval (25). During each contraction, the participant was instructed to gradually develop maximal strength over a few seconds and continue the maximal effort for 5 s (24). The maximal isometric force was converted into torque (Torque [N.m] = Force [kg] × leg length [m] × 9.81 [m/s2]) (25). The torques of both legs were summed to aggregate knee extensor torques (AggKET), then divided by body mass to be aggregate knee extensor torques normalised by body mass (AggKETbm, N.m/kg) (26).

## Statistical Analysis

The data analysis was performed with Statistical Package for Social Sciences (SPSS) version 17.0 (SPSS Inc., Chicago, IL, USA). The distribution of data was checked. If skewness or non-normality was identified, the natural log-transformation and back-transformation would be done (27–30). Geometric mean and 95% confidence interval (CI) was estimated by Cox’s modification (31–32).

## Reliability Analysis

Relative within-rater reliability was calculated using intra-class correlation coefficients (ICC_2,1_) with 95% CI for a two-way random effects model and absolute agreement (33). Lower one-sided 95% CI ICC_2,1_ was also computed and ≥ 0.70 was considered to be acceptable (12, 34). For non-normality data, Spearman’s correlation coefficient (SPC), with 95% CI and lower one-sided 95% CI, were calculated using Fisher’s transformation (35).

Absolute reliability included SEM, SEM percentage (SEM%), MDC and LOA. SEM was calculated as the square root of mean square error, and 95% CI was from sum square error and Chi-squared value from the ICC_2,1_ analysis of variance table (36). MDC_90_ was calculated from 1.65 × √2 × SEM. SEM% was defined as (SEM/mean) × 100 and MDC_90_ percentage (MDC_90_%) was (MDC_90_/mean) × 100, when mean was the mean of all observations in both sessions 1 and 2 (37). A SEM% of < 10% was the acceptable random error regardless of measurement unit (38). Coefficient of variation percentage (CV%) and 95% CI were calculated with the root mean square method (39). CV% < 10% was acceptable as the small difference lied within 10% of the mean (10). LOA was reported as LOA = mean_diff_ ± 1.96 (*Z*-score of 95% CI) × SD_diff_, when mean_diff_ and SD_diff_ were the mean and SD of the difference between sessions (40). LOA should cover ‘0’ to show that there was a point where between-session scores were equal. LOA was separated into systematic bias (left component or mean difference) and random error (right component or SD component). The systematic bias was interpreted as a percentage of the grand mean of the sample (10). For non-normality performance-based data, SEM (log scale), CV% and ratio LOA were analysed. Ratio LOA = mean_Lndiff_ + 1.96 × SD_Lndiff_, when Ln_diff_ was the difference of the log-transformed session 1 and 2 scores (Ln_s1_–Ln_s2_) (40). The log-form of ratio LOA was later back-transformed (antilog = e_s1/s2_) and reported as antilog (mean_Lndiff_) x/÷ antilog (1.96 × SD_Lndiff_) (14). Ratio LOA should cover ‘1’, which indicated equal between-session scores. For ratio LOA, the bias was interpreted as the percentage between repeated mean.

## Validity Analysis

The bivariate correlation coefficient was analysed among the performance-based test, KOOS-PS, normalised AggKET, NRS pain and age. Pearson’s correlation coefficient (for normality data), SPC (for non-normality data and ordinal scale) and their 95% CI was calculated using Fisher’s transformation (35). For convergent validity, the relationship between performance test and the measurement with similar constructs (KOOS-PS, knee extension torque) should have a correlation coefficient at least moderate, ≥ 0.4 or ≤ −0.4 (13). All performance tests were evaluated for known-groups validity using adaptation to stair climbing as an independent variable. The variable was categorised into two groups; non-adaptation and adaptation (e.g. use of walking aids or handrail). Subgroup comparison of each performance measure was done using *t*-test or univariate Analysis of Covariance (ANCOVA) if any covariates were detected. ANCOVA was perform with standard procedures and no violation of assumption; i.e. homogeneity of regression slope (*P*-value for *F_interaction_* > 0.05), homogeneity of variance (*P*-value for Levene’s test > 0.05) and variance ratio (*F_max_* ≥ 10:1) (27, 41). Main effect *F*-test, estimated marginal mean (statistical covariate adjustment), partial eta squared effect size (Eta^2^) with 95% CI (non-centrality interval estimation) were calculated (41–43). Cohen’s criteria for Eta^2^ was 0.01 (1%) small effect, 0.06 (6%) medium and 0.138 (14%) large (41).

## Results

**Figure 1:**
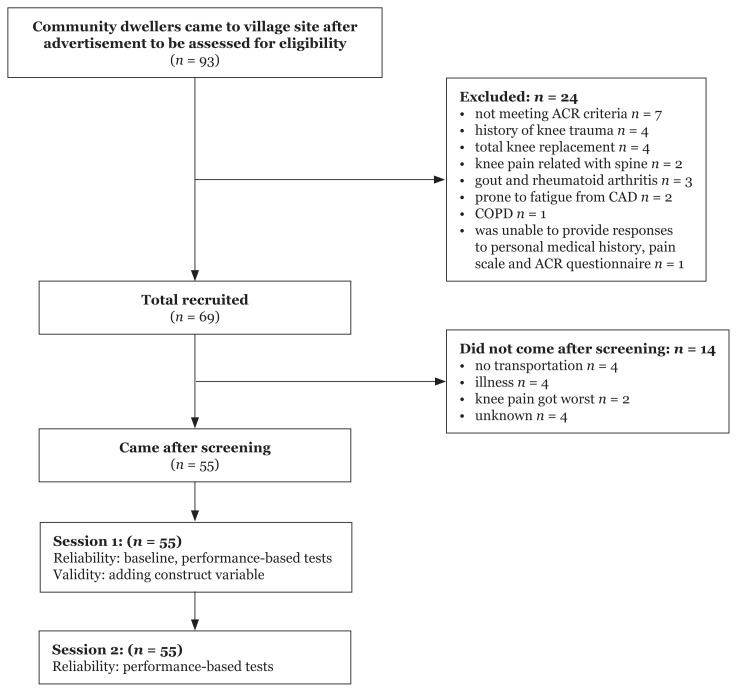
Number of patients excluded and included and reasons for not being included in the study

Ninety-three community dwellers came to the village sites and screened using the ACR and eligibility criteria. Of those, 24 were excluded who did not meet ACR criteria (*n* = 7), had history of knee trauma (*n* = 4) and total knee replacement (*n* = 4), had knee pain related with spinal conditions (*n* = 2), other knee pathology, i.e. gout (*n* = 1) and rheumatoid arthritis (*n* = 2), were prone to fatigue and weak from coronary artery disease (*n* = 2), had chronic obstructive pulmonary disease (*n* = 1) and was unable to provide responses to personal medical history, pain scale and ACR questionnaire (*n* = 1). Of the remaining 69, 14 did not come after the screening session because of no transportation (*n* = 4), illness (*n* = 4), knee pain got worst (*n* = 2) and unknown (*n* = 4). Finally, 55 participants joined both sessions 1 and 2 of the data collection, and their complete data were available. The number of patients included and reasons for not being included in the study are summarised in Figure 1. Participant characteristics are presented in Table 1. All variables were normally distributed, except SCT, age and body mass. Only 9-step SCT was natural log-transformed for further analysis. Their skewness and kurtosis were improved to be normal after transformation without the outlier.

### Between Sessions 1-Week Interval Within-Rater Reliability

Descriptive data and within-rater absolute and relative reliability of three performance-based tests are presented in Table 2. In terms of relative reliability, all performance-based tests were well above acceptable levels (ICC and SPC > 0.85, lower 1-sided 95% CI > 0.7). Of all absolute reliability, SEM% and MDC% could be interpreted for normality data, i.e. 30sCST and 40mFPWT. SEM% of both tests was well under 10% (9.1% and 7.0%) of the mean test score and showed a small amount of random error. For CV%, the 40mFPWT and 9-step SCT achieved an acceptable level of <10% (6.9% and 6.7%, respectively), whilst the 30sCST was 0.7% above the criteria (10.7%). The LOA of both 30sCST and 40mFPWT showed small systematic bias (−0.9 times and −0.002 m/s, or 6.1% and 0.2% of the grand mean, respectively) and covered ‘0’, meant that between sessions test scores were sometimes equal. The difference between session test scores of 30sCST and 40mFPWT, with 95% CI, lied within 3.8 times and 0.245 m/s, respectively. For ratio LOA, the 9-step SCT showed a ratio of 1.029 of systematic bias, which signified a 2.9% difference between session test scores, and a ratio of 1.188 of random error, which signified with 95% CI, no more than 18.8% of the difference.

### Construct Validity

Bivariate correlation coefficients of the tested variables are shown in Table 3. Of three similar constructs, only AggKETbm met the minimum acceptable level of ≥ 0.4 or ≤ −0.4 with all performance-based tests ([30sCST, 0.41]; [40mFPWT, 0.50]; [9-step SCT, −0.42]). The KOOS-PS and KOOS-PS question 3 showed significant correlation only with 30sCST (*r* = −0.27 and *r* = −0.30) but did not meet the minimum acceptable criteria. The ‘absolute’ correlation between KOOS-PS and NRS pain was higher than the correlation between all performance tests and NRS pain of at least 0.1 ([30sCST, 0.27/−0.05]; [40mFPWT, 0.27/−0.17]; [9-step SCT, 0.27/0.12]).

### Known-Groups Validity

Since age was significantly (*P* < 0.01) related with all performance-based tests (30sCST, *r* = −0.48; 40mFPWT, *r* = −0.37; 9-step SCT, *r* = −0.55) ANCOVA adjusted for age was performed to compare the performance ability between non-adaptation and adaptation (during stair climbing) groups. The results of ANCOVA are presented in Table 4. All, except 9-step SCT model, showed no assumptions violation. Although the Levene’s test demonstrated unequal variance, the *F_max_* was less than 10:1, and near-equal sample size between cells (*n* = 27 and 28) supported robust ANCOVA analysis. With statistical age-adjustment, the non-adaptation group had better performance than the adaptation group significantly with mean difference of 2.6 stands of 30sCST, 0.161 m/s of 40mFPWT and 49.2% of 9-step SCT. The percentage of variance in the DV (performance test scores), as explained by the IV (adaptation of 9-step SCT), was large in the 9-step SCT (40.6%) and 40mFPWT (16.3%) and medium in 30sCST (12.1%).

## Discussion

This study aimed to evaluate the psychometric properties of the OARSI recommended minimal core set of three performance-based tests, including various forms of reliability and construct validity. In terms of reliability, all tests showed acceptable levels of relative reliability and small measurement error. For convergent validity, the tests showed moderate correlation with age and knee extensor torque but low correlation or no relationship with self-reported physical function. In terms of known-group validity, all tests could discriminate between the use and non-use stair-climbing aids groups.

In terms of relative reliability, 9-step SCT showed better consistency than the other two tests. To compare the degree of absolute reliability from a different study, SEM% is an accurate estimation for homoscedasticity and CV% for heteroscedasticity. To determine whether the change of intervention is real, MDC% is helpful for homoscedastic data. For heteroscedastic data, ratio LOA could be a minimum criterion of change (10, 14). In this study, the ratio of repeated 9-step SCT test time should be at least 18.8% (with 95% CI). The relative reliability (lower 1-sided ICC) of this study was within the same range as other studies (12, 44). However, the absolute reliability based on MDC90%, in this study (30sCST, 21.1%; 40mFPWT, 16.3%), showed little more error than those of previous study (30sCST, 16.9%; 40mFPWT, 9.5%). For 9-step SCT, MDC90% of the previous study (18.8% of the average mean) was comparable with the random error of ratio LOA in this study (18.8% of the ratio between-session mean). The mean 9-step SCT time of this study (14.2 s and 14.7 s) was higher than that in a previous study (12) (13.27 s and 12.35 s). Age factor may conjunct the performance of this knee OA group and contribute to higher random error.

For convergent validity, AggKETbm was the only construct that passed the minimum acceptable criteria. The self-report physical function (KOOS-PS) did not show significant correlation with all the performance-based tests except 30sCST. However, the KOOS-PS and 30sCST correlation coefficient were less than the criteria. The ‘absolute’ correlation between KOOS-PS and NRS pain was higher than the correlation between all performance tests and NRS pain. Since self-report and performance-based tests were developed to assess similar function in OA (3) (e.g. ICF conceptualisation), it was expected to have a meaningful association. The result of this study and the previous study did not respect this premise. These unmet criteria of the correlation might give the idea of the content validity of both self-reported and performance-based tests. Timed measurement of task performance is the reduction of direct observation to be one quantifiable dimension, which dismisses erroneous movement (i.e. impairment) (45–46). It was advocated that the assessed content of the self-report was not only the patient’s ability to move around but also the patient’s subjective response to accumulated past experiences (i.e. pain and perceived exertion) (45). The results in the influence of pain on the self-report were more than the pain experienced upon the execution of that task (13, 45, 47), as showed in this study.

In this study, the failure of the three performance-based tests to show hypothesised relationship with KOOS-PS and NRS might be the problem of construct under representation (48). If a measured test underrepresents the physical component function, it fails to capture the important aspects of the construct it purports to measure. In this study, the correlation between 30sCST and K-PS-Q3 or the question number 3 was more than the correlation with KOOS-PS, which also suggested that 30sCST was more strongly represented by a component of KOOS-PS.

For known-group validity, this study used stair climbing aids (use/non-use) as a discriminatory factor, which was differed from the previous study (gait aid/no gait aid) (16). Since age was correlated well with all performance-based tests and also showed a significant difference between use and non-use stair climbing aids (not presented in the results; independent *t*-test = −2.763, *P* = 0.008), the age was adjusted to accurately estimate the effect size. All three performance-based tests demonstrated evidence of known-groups validity by differentiation of the knee OA group with aids from the group without aids. The 9-step SCT showed a larger effect size than the other two tests. It suggested that 9-step SCT was more relevant to adaptation of stair climbing factor than 30sCST and 40mFPWT.

In this study, heterogeneity of data and skewness were observed in the stair-climbing performance test. Natural log was used to transform because of easy-interpretation when back-transformation as stated above. The Cox estimation of 95% CI was selected because of its smallest coverage error for a medium sample size (31). With our knowledge, this is the first study to estimate various absolute reliability of recommended performance-based tests, specifically for different data types (e.g. homogenous and heterogeneous). Since some human performance measurement was recorded in a ratio scale and lead to heteroscedastic errors (14), the CV% and ratio LOA were more appropriate. A 1-week duration was set to ensure between-session within-rater reliability rather than test–retest reliability, as other studies (13, 49). The sources of heteroscedasticity in 9-step SCT seemed to relate with the level of adaptation whose division made a large effect size. Age factor was the confounder for construct validity of these performance-based tests. According to the Thailand context of knee OA burden in community-dwelling elderly (22.5% in 2014) (50), the combined effects of sarcopenia and arthrogenically reduced voluntary activation mechanism to strength and function deterioration which should be considered (2, 51–53).

This study had several limitations. First, the study design was cross-sectional and scope on the change of repeated measurement rather than responsiveness of the change from intervention. Second, although the proposed sample size of > 50 is adequate (17), the heteroscedastic data might need more sample size than ‘adequate’. Third, KOOS-PS had narrow valid content. It showed an inferior ability to assess lower extremity function than KOOS functional and Sports items sum scores (54). The reduction of items might result in the under representation of KOOS-PS. Lastly, the measures used for convergent validation might well selected on other relevant outcomes such as quality of life, pain associated with function.

## Conclusion

This study evaluated a number of selected psychometric properties of the OARSI recommended minimal core set of performance-based tests in knee OA. The reliability and measurement error estimated all tests to meet the acceptable criteria. MDC90 and random error component of ratio LOA were calculated for useful decision of real change. The convergent validity only with knee extensor strength demonstrated limited representation. The performance-based tests had medium to large effect size in discriminating the group adaptation to stair climbing. The study suggested using specific calculation of absolute reliability to homo-/heterogenic data.

## Figures and Tables

**Table 1:** Baseline characteristics of the participants (*n = 55*)

Variables	Mean (SD)	*n* (%)
Age (years)^a^	69.0 (11)	
Sex		
Male		18 (22.7)
Female		37 (67.3)
Height (m)	1.54 (0.09)	
Body mass (kg)^a^	50.0 (15)	
BMI groups		
Underweight (BMI < 18.50)		10 (18.2)
Healthy weight (BMI 18.50 – 22.99)		23 (41.8)
Overweight (BMI 23.00–24.99)		5 ( 9.1)
Obese (BMI ≥ 25.00)		17 (30.9)
OA side involved		
Unilateral (right)		11 (20.0)
Unilateral (left)		11 (20.0)
Bilateral		33 (60.0)
NRS pain^a^	5 (3)	
KOOS-PS	42.13 (11.159)	
AggKET (N.m)	105.91 (30.265)	
AggKETbm (N.m/kg)	2.01 (0.519)	

Note: BMI = body mass index; SD = standard deviation; OA = osteoarthritis; NRS = numerical rating scale; KOOS-PS = Knee Injury and Osteoarthritis Outcome Score-Physical Function Short Form; AggKET = aggregate knee extensor torque; AggKETbm = aggregate knee extensor torque; normalised by body mass;

a median (interquartile range = IQR)

**Table 2:** Between sessions reliability and measurement errors (*n* = 55)

	30sCST	40mFPWT (m/s)	9-step SCT(s)	
	
	(no of stands)		Ln	back-transformed
Session 1 mean (SD)	14.6 (4.1)	1.195 (0.228)	2.685 (0.345)	14.7 (13.3, 16.1)^a^
Session 2 mean (SD)	15.6 (4.2)	1.198 (0.204)	2.656 (0.339)	14.2 (13.0, 15.6)^a^
ICC (95% CI)	0.87 (0.74, 0.93)	0.85 (0.75, 0.91)		0.92 (0.87, 0.95)^b^
Lower 1-sided 95% CI	0.77	0.77		0.88^b^
SEM (95% CI)	1.4 (1.2, 1.7)	0.084 (0.072, 0.105)	0.062 (0.052, 0.076)	1.064 (1.054, 1.079)
SEM%	9.1	7.0	–	–
MDC_90_ (%)	3.2 (21.1)	0.2 (16.3)	–	–
CV% (95% CI)	10.7 (7.45, 13.2)	6.9 (5.1, 8.4)		6.7 (6.0, 7.3)
LOA	−0.9 ± 3.8	−0.002 ± 0.245	0.029 ± 0.172	1.029 x/÷ 1.188^c^
95% CI	−4.7, 2.9	−0.248, 0.243	−0.143, 0.201	0.867, 1.222^c^

Notes: 30sCST = 30-s chair-stand test; 40mFPWT = 40-m fast-paced walk test; 9-step SCT= 9-step stair climb test; Ln = natural log transformation; back-transformed = antilog or exponent; ICC = intra-class correlation coefficient; 95% CI = 95% confidence interval; SEM = standard error of measurement; MDC_90_ = minimum detectable change at the 90% CI level; CV% = coefficient of variation percentage; LOA = limit of agreement;

a geometric mean (95% CI: lower limit, upper limit);

b Spearman’s correlation coefficient;

c ratio LOA

**Table 3:** Bivariate Pearson’s correlation coefficient (95% CI)( *n* = 55)

	30sCST	40mFPWT	9-step SCT^a^	KOOS-PS	K-PS-Q3^a^	NRS^a^	AggKETbm
30sCST							
40mFPWT	0.57** (0.35, 0.72)						
9-step SCT^a^	−0.60** (−0.39, −0.74)	−0.64** (−0.45, −0.77)					
KOOS-PS	−0.27* (−0.01, −0.50)	−0.22 (−0.46, 0.05)	0.23 (−0.04, 0.47)				
K-PS-Q3^a^	−0.30* (−0.03, −0.52)	−0.24 (−0.47, 0.03)	0.25 (−0.02, 0.48)	0.57** (0.35, 0.72)			
NRS^a^	−0.05 (−0.31, 0.22)	−0.17 (−0.41, 0.10)	0.12 (−0.15, 0.37)	0.31* (0.04, 0.53)	0.34* (0.08, 0.56)		
AggKETbm	0.41** (0.16, 0.61)	0.50** (0.28, 0.68)	−0.44** (−0.20, −0.63)	−0.28* (−0.01, −0.51)	−0.29* (−0.02, −0.51)	0.01 (−0.26, 0.28)	
Age^a^	−0.48** (−0.24, −0.66)	−0.37** (−0.11, −0.58)	0.55** (0.34, 0.71)	−0.06 (−0.32, 0.21)	0.09 (−0.18, 0.35)	−0.01 (−0.27, 0.26)	−0.28* (−0.01, −0.50)

Notes:

** significant at *P* < 0.01,

* significant at *P* < 0.05

30sCST = 30-s chair-stand test; 40mFPWT = 40-m fast-paced walk test; 9-step SCT = 9-step stair climb test; KOOS-PS = knee injury and osteoarthritis outcome score-physical function short form; K-PS-Q3 = KOOS-PS question no. 3 (rising from sitting); AggKETbm = aggregate knee extensor torque normalised by body mass;

a Spearman’s correlation coefficient

**Table 4:** Analysis of covariance on three performance-based tests (DV) adjusted for covariate (*n* = 55)

DV	Estimated mean (SE)		Interaction *F_1,51_* (*P*-value)	Levene *F_1,53_* (*P*-value)	Var_R_	Main effect *F_1,52_* (*P*-value)	Eta^2^ (95%CI)^a^

	Ad. (*n* = 27)	Non-Ad. (*n* = 28)					
30sCST	13.30 (0.670)	15.89 (0.657)	0.119	0.610	1.066	7.139	0.121
(no. of stands)			(0.732)	(0.438)		(0.010)	(0.007, 0.289)
40mFPWT	1.12 (0.035)	1.28 (0.034)	0.070	1.924	1.413	10.159	0.163
(m/s)			(0.792)	(0.171)		(0.002)	(0.023, 0.336)
9-step SCT^b^	2.80 (0.046)	2.40 (0.046)	0.024	15.666	3.565	35.548	0.406
(s)			(0.877)	(< 0.001)		(< 0.001)	(0.200, 0.555)

Notes: DV = dependent variable; estimated mean (SE) = estimated marginal mean and standard error of the mean by covariate (age) adjustment at 71.20 years; Ad. = stair climb with adaptation; Non-Ad. = stair climb without adaptations; interaction = test of homogeneity of regression or interaction between covariate (age) and independent variable (Ad./Non-Ad.); Levene = Levene’s test of homogeneity of variance; Var_R_ = variance ratio or F_max_; main effect = univariate ANCOVA between effect; Eta^2^ = partial eta squared or effect size; 30sCST = 30-s chair-stand test; 40mFPWT = 40m fast-paced walk test; SCT = 9-step stair climb test;

a Non centrality interval estimation,

b Natural log transformation
